# Biased virus transmission following sequential coinfection of *Aedes aegypti* with dengue and Zika viruses

**DOI:** 10.1371/journal.pntd.0012053

**Published:** 2024-04-01

**Authors:** Jiameng Peng, Meichun Zhang, Gang Wang, Dongjing Zhang, Xiaoying Zheng, Yongjun Li

**Affiliations:** 1 Department of Pathogen Biology, School of Medicine, Jinan University, Guangzhou, Guangdong, China; 2 Key Laboratory of Tropical Disease Control (Sun Yat-sen University), Ministry of Education, Guangzhou, Guangdong, China; WRAIR, UNITED STATES

## Abstract

**Background:**

Mosquito-borne arboviruses are expanding their territory and elevating their infection prevalence due to the rapid climate change, urbanization, and increased international travel and global trade. Various significant arboviruses, including the dengue virus, Zika virus, Chikungunya virus, and yellow fever virus, are all reliant on the same primary vector, *Aedes aegypti*. Consequently, the occurrence of arbovirus coinfection in mosquitoes is anticipated. Arbovirus coinfection in mosquitoes has two patterns: simultaneous and sequential. Numerous studies have demonstrated that simultaneous coinfection of arboviruses in mosquitoes is unlikely to exert mutual developmental influence on these viruses. However, the viruses’ interplay within a mosquito after the sequential coinfection seems intricated and not well understood.

**Methodology/principal findings:**

We conducted experiments aimed at examining the phenomenon of arbovirus sequential coinfection in both mosquito cell line (C6/36) and *A*. *aegypti*, specifically focusing on dengue virus (DENV, serotype 2) and Zika virus (ZIKV). We firstly observed that DENV and ZIKV can sequentially infect mosquito C6/36 cell line, but the replication level of the subsequently infected ZIKV was significantly suppressed. Similarly, *A*. *aegypti* mosquitoes can be sequentially coinfected by these two arboviruses, regardless of the order of virus exposure. However, the replication, dissemination, and the transmission potential of the secondary virus were significantly inhibited. We preliminarily explored the underlying mechanisms, revealing that arbovirus-infected mosquitoes exhibited activated innate immunity, disrupted lipid metabolism, and enhanced RNAi pathway, leading to reduced susceptibility to the secondary arbovirus infections.

**Conclusions/significance:**

Our findings suggest that, in contrast to simultaneous arbovirus coinfection in mosquitoes that can promote the transmission and co-circulation of these viruses, sequential coinfection appears to have limited influence on arbovirus transmission dynamics. However, it is important to note that more experimental investigations are needed to refine and expand upon this conclusion.

## Author summary

Arboviruses coinfection in mosquitoes can occur either simultaneously or sequentially. Simultaneous coinfection of arboviruses in mosquitoes is unlikely to exert mutual developmental influence on these viruses, resulting in a single mosquito bite may transmit multiple arboviruses to humans, thereby facilitating the spread of these pathogens. However, the interplay between viruses within a mosquito after the sequential coinfection is intricated and not well understood. We present results from DENV (serotype 2) and ZIKV sequential coinfection both in mosquito cell line (C6/36) and *Aedes aegypti*. Our results demonstrated that both mosquito cells and adult mosquitoes could be sequentially coinfected, but prior infection with one arbovirus significantly reduced susceptibility to a secondary arbovirus infection. Moreover, upon sequential coinfection with DENV and ZIKV through oral feeding, the transmission potential of *A*. *aegypti* for the ZIKV was aborted. We preliminarily explored the underlying mechanism of this phenomenon and observed that arbovirus-infected mosquitoes exhibited activated innate immunity, disrupted lipid metabolism, and enhanced RNAi pathway, all of which contributed to the reduced susceptibility to the secondary arbovirus infections. In the epidemiology of mosquito borne diseases, our findings indicates that, in contrast to simultaneous arbovirus coinfection in mosquitoes that can promote the transmission and co-circulation of these viruses, sequential coinfection appears to have limited influence on arbovirus transmission dynamics.

## Introduction

Dengue virus (DENV) and Zika virus (ZIKV) are taxonomically classified within the genus *Flavivirus*, exhibiting a genetic homology over 90% in their genomic sequences [1]. The resulting diseases attributed to these viruses are imposing substantial and escalating health challenges upon global human populations. DENV threatens nearly half of the world’s population, causing an estimated 100–400 million infections annually [2]. The risk of dengue transmission is further exacerbated by the rapid pace of climate change and urbanization. These factors not only result in the expanded geographical distribution of *Aedes* mosquitoes [3–5] but also enhance the mosquito’s vectorial capacity for dengue virus transmission [6,7], thereby increasing the vulnerability of communities to dengue outbreaks [4]. ZIKV was first isolated in 1947 from a febrile rhesus monkey in the Zika forest of Uganda [8]. For the subsequent half century, human infections were sporadic and presented with mild symptoms. However, major Zika pandemics emerged from 2015 to 2016 [9], and garnered global concern due to the severe neurological abnormalities, including neonatal microcephaly and Guillain-Barré syndrome, associated with ZIKV infection [10–14]. Despite the subsiding of the Zika pandemic since 2017, the threat of ZIKV persists due to several factors. Firstly, the majority of populations remain susceptible to ZIKV infection, and there is a potential for antibody-dependent enhancement (ADE), which can be facilitated by pre-existing immunity to other related viruses such as DENV or chikungunya virus (CHIKV) [15–19]. Secondly, ZIKV is not disappearing but continues to maintain a low level of prevalence in certain regions [20–22]. Lastly, occasional occurrence of mutations in ZIKV raises concerns as these mutations may enhance its adaptation in the human host and potentially lead to more severe infections [19,23–25]. Unfortunately, at present, there are no available vaccines or highly effective therapeutic drugs for the prevention or treatment of DENV or ZIKV infections.

DENV and ZIKV are primarily transmitted by *Aedes aegypti*, and their epidemic regions largely overlap [26,27], providing an opportunity for coinfection of DENV and ZIKV in *A*. *aegypti* [28,29]. Although direct evidence of arbovirus coinfection in field-caught mosquitoes is rare [30–32], multiple studies have demonstrated that *A*. *aegypti* can undergo simultaneous infection with various arboviruses including DENV, CHIKV, and ZIKV, and subsequently transmit them in a single bite [29,33–35]. Throughout the Zika pandemic, there were frequent reports of coinfection cases involving DENV and ZIKV [36–39]. Although patients coinfected with arboviruses typically experienced mild symptoms [27,40,41], fatal cases have also been reported in patients coinfected with CHIKV and DENV [42]. Since all viruses may be present in the serum of patients coinfected with multiple arboviruses [43,44], mosquitoes feeding on these patients can ingest multiple viruses, potentially resulting in simultaneous coinfection. Extensive investigations into simultaneous arbovirus coinfection in mosquitoes have consistently shown that coinfected arboviruses do not negatively interfere with each other’s transmission [29,33,35,43–45], which implies that a single mosquito bite has the potential to transmit two or more arboviruses simultaneously. As a result, simultaneous coinfection of arboviruses in mosquitoes is likely to enhance their co-transmission, thereby contributing to the spread and extent of epidemics.

In regions where multiple arboviruses co-circulate, mosquitoes can experience sequential coinfection when they bite individuals infected with different arboviruses during a single or multiple gonotrophic cycles. Sequential coinfections are more probable compared to simultaneous coinfections of arboviruses [46]. The interaction between these viruses in sequential coinfection may differ from that observed during simultaneous coinfection, potentially resulting in altered patterns of virus transmission. Research conducted on mosquito cell lines consistently showed that prior infection with one arbovirus can restrict or even prevent subsequent arbovirus infection [47–50]. However, there is a limited number of studies that have investigated the phenomenon of arbovirus sequential coinfection in mosquitoes and have yielded varied findings. For instance, when *A*. *aegypti* mosquitoes were infected with DENV-2 either before or after infection with the Murray Valley encephalitis virus, the replication and transmission of the latter virus were unaffected [51]. When *A*. *albopictus* mosquitoes sequentially infected with DENV-1 and CHIKV, both virus particles exhibited in head squashes [52]. Another study indicated that initial infection with DENV-2 or CHIKV did not impact subsequent ZIKV replication and infection rates in *A*. *aegypti* head squashes [53]. These results suggested that the presence of one arbovirus in *Aedes* mosquitoes did not appear to significantly influence the development of a secondary infecting virus. In contrast, a study on *Culex quinquefasciatus* sequentially co-infected with West Nile virus (WNV) and St Louis encephalitis virus (SLEV) demonstrated that prior exposure to one virus reduced susceptibility and dissemination of the other [54]. Similar virus interference was observed when *A*. *aegypti* sequentially coinfected with DENV of different serotype [55]. Furthermore, a study showed that sequential coinfection of *A*. *aegypti* with CHIKV and ZIKV had enhanced the transmission potential of ZIKV [56]. In summary, it appears that the arboviruses sequential co-infection in mosquitoes implies a more complex interaction among these viruses. Consequently, knowing the interactions of diverse arboviruses within a mosquito vector is crucial for comprehending the potential influence of arbovirus sequential coinfection on the epidemiology of these arboviruses in their co-endemic areas.

In this study, we conducted experiments aimed at examining the phenomenon of arbovirus sequential coinfection in both mosquito cell line (C6/36) and *A*. *aegypti*, specifically focusing on dengue virus (DENV, serotype 2) and Zika virus (ZIKV). We firstly observed that DENV and ZIKV can sequentially infect mosquito C6/36 cell line, but the replication level of the subsequently infected ZIKV was significantly suppressed. Similarly, *A*. *aegypti* mosquitoes can be sequentially coinfected by these two arboviruses, regardless of the order of virus exposure. However, the replication and dissemination of the secondary virus were significantly inhibited, and more importantly, the transmission potential of the secondary virus was nearly aborted. Furthermore, our results indicate that the subsequent ZIKV infection did not impact the prior development of DENV, while the subsequent DENV infection resulted in reduced replication of prior ZIKV. We also preliminarily explored the underlying mechanisms, revealing that arbovirus-infected mosquitoes exhibited activated innate immunity, disrupted lipid metabolism, and enhanced RNAi pathway, leading to reduced susceptibility to the secondary arbovirus infections. These findings suggest that, in contrast to simultaneous arbovirus coinfections that can promote the transmission and co-circulation of these viruses, sequential coinfection appears to exert limited influence on arbovirus transmission dynamics.

## Methods

### Ethics statement

Ethical approvals for this study were obtained from the Ethics Committee of Zhongshan School of Medicine (approval number 2017–041) and Jinan University Laboratory Animal Ethics Committee (approval number 20220220–01).

### Mosquito colony maintenance

*Aedes aegypti* (Guangdong Line) was collected in Guangdong Province and kindly provided by Guangdong Provincial Center for Disease Control and Prevention (CDC). This colony was maintained in a BSL-2 insectary at 27±2°C and 75%-85% humidity with a 12-hr light/dark cycle. Larvae were fed with bovine liver powder. Adult mosquitoes were provided with 10% sucrose solution. Female mosquitoes were fed on a mouse to lay eggs.

### Virus culture and titration

DENV (serotype 2, New Guinea C strain) was cultured and titrated in C6/36 cells as previously described with minor modification [57]: DENV was inoculated into C6/36 cells and incubated for 5 days under 35°C, 5% CO_2_. Then virus was collected and immediately titrated. The serially diluted virus was inoculated into C6/36 cells in 6 well plates, and then cells were covered with 2% methylcellulose (2g methylcellulose dissolved in 100ml Dulbecco’s modified eagle medium (DMEM) containing 2% fetal bovine serum (FBS)). After incubation for 5 days at 35°C and 5% CO_2_, cells were fixed with 4% paraformaldehyde and then stained with hematoxylin. Plaques were counted under a microscope. ZIKV strain Z16006 was initially isolated from a patient in 2016 (provided by Guangdong CDC). In contrast to the cytopathic effect (CPE) observed in C6/36 cells during DENV infection, no apparent CPE was seen during ZIKV infection. As a result, the plaque-forming units of ZIKV were visualized using an immunohistochemical staining technique. Briefly, after 5 days of incubation in C6/36 cells at 35°C and 5% CO_2_, cells were fixed with 4% paraformaldehyde, washed with PBS and treated with 0.5% triton X-100 in phosphate buffer saline (PBS) for 20 minutes under room temperature. The cells were then incubated with 3% H_2_O_2_ for 10 minutes and blocked by PBS with 5% normal goat serum and 0.3% triton X-100 for 1 hour under room temperature. The primary antibody for ZIKV (BioFront) was 1:500 diluted in PBS and then added to each well, incubated at 37°C for 1 hour. After 3 times TBST rinse, 100μl SignalStain boost IHC detection reagent (Cell Signaling Technology) was added to each well and incubated at room temperature for 30 minutes. After TBST rinse, DAB was added to each well to visualize the plaques.

### Virus sequential coinfection in mosquito cells

C6/36 cells were grown in 24 well plates until reach a ~100% confluency. Then DENV was inoculated into cells at an MOI of approximately 0.01. Twelve hours later, ZIKV was inoculated into the DENV-infected cells at a MOI of approximately 0.01. Each treatment was sampled in 4 wells at 12-hour intervals over the course of 6 periods (from 12 hours to 72 hours post coinfection). C6/36 cells infected only with ZIKV were used as a control to compare ZIKV growing kinetics with and without previous DENV infection. C6/36 cells only infected with DENV also served as another control to study if later ZIKV infection could have any influence on previous DENV infection. Virus growing kinetics was analyzed by RT-qPCR (see below) and presented as virus RNA copies per *rps6*.

### Virus visualization in cells

A double immunofluorescence experiment was performed to visualize DENV and ZIKV coinfection in C6/36 cells. Twelve hours post DENV inoculation, ZIKV was inoculated into C6/36 cells, then they were incubated for 24 hours under 35°C, 5% CO_2_. Cells were fixed with 4% paraformaldehyde for 10 minutes under room temperature. After PBS rinse, cells were incubated with block solution for 1 hour under room temperature. Primary antibody for DENV (rabbit anti-dengue, 1:200, Abcam) and ZIKV (mouse anti-ZIKV, 1:500, Biofront) was mixed and added onto cells, incubated overnight at 4°C. After PBS rinse for 3 times, secondary antibodies, goat anti-rabbit (labeled with Alexa Fluor 555, Cell Signaling Technology) and goat anti-mouse (labeled with Alexa Fluor 488, Cell Signaling Technology), were diluted at 1:500, and then added onto cells, incubated for 2 hours under room temperature. Cells were then washed with PBS and stained with 4’,6-diamidino-2-phenylindole (DAPI) (Roche) for 5 minutes. Images of different stained specimens were captured using an Olympus IX70 fluorescence microscope and then merged using the microscope’s included software.

### Virus sequential coinfection in mosquitos

For experiments involving sequential coinfection of *A*. *aegypti* with DENV followed by ZIKV, mosquitoes were infected either through intrathoracic inoculation or oral feeding. For intrathoracic inoculation, 69 nl of -80°C preserved DENV (virus copy number: 10^5.5^/ml) supernatant was firstly injected into approximately 30 female mosquitoes of 2–3 days old, using a Nanoject II microinjector (Drummond). Seven days post DENV infection, ZIKV (virus copy number: 10^6.3^/ml) was inoculated into the survivors. Then another 7 days later, the survived mosquitoes were collected for vector competence study (see below). In this experiment, two control groups were set: mosquitoes were firstly injected with DMEM and then ZIKV (group: DMEM+ZIKV), or mosquitoes were firstly injected with DENV and then DMEM (group: DENV+DMEM). For virus oral infection, procedures were proceed as previously described [57]. Briefly, approximately a hundred 6–7 days old mosquitoes were allowed to feed on a blood meal mixed with equal volume DENV supernatant (final virus titer at 10^7.18^ PFU/ml) for 30–45 minutes through a water bath circulation system (Fisher). Engorged mosquitoes were selected and reared under the conditions mentioned above, and provided with oviposition sites for laying eggs 3 days post blood-feeding. Those mosquitoes were provided with the second blood meal mixed with ZIKV supernatant (final virus titer at 10^6.90^ PFU/ml) 7 days later. Engorged mosquitoes were kept for subsequent research. Also, an artificial breeding site was provided three days after the second blood-feeding. Throughout the experiment, all eggs produced by the virus-infected females were discarded after autoclaving. In this experiment, only one control group was set: mosquitoes were firstly fed with DMEM and then ZIKV (group DMEM+ZIKV). Sheep blood was used in virus oral-infection. For experiments involving sequential coinfection of *A*. *aegypti* with ZIKV followed by DENV, only intrathoracic inoculation was conducted. Approximately 30 mosquitoes were firstly injected with ZIKV and then the survivors were inoculated with DENV 7 days later. In this experiment, ZIKV copies were at 10^5.4^/ml, and DENV copies were at 10^5.9^/ml. Virus supernatants were stored at -80°C before use. There were two control groups for this experiment: mosquitoes were firstly injected with DMEM and then DENV (group DMEM+DENV), or mosquitoes were firstly injected with ZIKV and then DMEM (group ZIKV+DMEM).

### Vector competence assay

After the virus infection, tissues were collected and homogenized, total RNA was extracted and reverse transcribed, and viral RNA was detected (see below). Virus replication and dissemination were assessed by viral RNA copies in the body (thorax and abdomen) and head/legs, respectively. For transmission assay, saliva samples were collected using the forced salivation technique as described previously with minor modification [58,59]. Briefly, Mosquitoes were knocked down by CO_2_ and their legs and wings were removed, then the mosquito proboscis was inserted into a 10 μl pipette tip containing 6 μl of FBS and kept for 30–45 minutes under room temperature. The number of copies of DENV-2 *NS5* or ZIKV *NS1* in the saliva was quantified by RT-qPCR as described below.

### RNA extraction and virus quantification

Total RNA was extracted by RNAiso reagent (Takara) according to the manufacturer’s protocol. RNA was dissolved in RNase-free water. The first strand of cDNA was synthesized using HiScript II Q SuperMix for qPCR (+gDNA wiper) (Vazyme). Virus RNA was quantified by qPCR on the LightCycler96 Detection System (Roche) using TB Green Premix Ex Taq II (Tri RNaseH Plus) (Takara), with the following conditions: 95°C for 30 s, then 40 cycles of 90°C for 5 s and 60°C for 30 s, followed by melting curve analysis. For virus quantification, genes of DENV *NS5* or ZIKV *NS1* and mosquito *rps17* were separately cloned into the pMD-18T vector (Takara) separately as described previously [59,60]. Serial dilution of the above recombinant plasmids was used as a standard to generate standard curves. Virus copy numbers in mosquito tissues were normalized by *rps17*. Primers for DENV-2 *NS5* [61], ZIKV *NS1* [62], *rps6* [62], and *rps17* [63] have previously been reported.

### Expression level of immune and lipid synthesis related gene

Six to seven days old female *Ae*. *aegypti* were injected with either DENV-2 or ZIKV as described above. Mosquitoes injected with PBS were served as control groups. Twenty-four hours post-injection, the mosquitoes were collected and killed under -20°C, 8 to 10 mosquitoes were pooled together for RNA extraction and immune-related gene mRNA expression quantification. Primers for *CECG* [64], *DEFA* [64] and *Dcr2* [65] were previously reported. Primers for *Fas1* was newly designed: forward 5’- CAAGCCGTACCAAGCCTACA -3’, reverse 5’- ATCCAGATGCTTGGTCGCAA -3’.

### Statistical analysis

Statistical comparisons of virus load in cells, tissues, and saliva samples were conducted using the two-tailed Mann-Whitney U test. Significance of virus prevalence in mosquito saliva samples was determined by Fisher’s exact test. Statistical comparisons of relative mRNA expression levels in mosquitoes following different treatments were assessed using the one-way ANOVA with Bonferroni correction. Data statistical analysis was done by using IBM SPSS statistic 20 and GraphPad Prism 6.0 software.

## Results

### ZIKV replication is inhibited in previously DENV infected C6/36 cells

**Fig 1:**
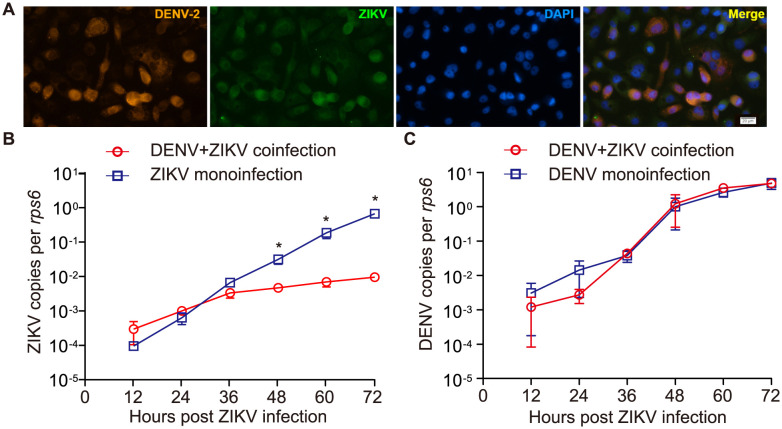
Visualization and growth kinetics of sequential DENV and ZIKV coinfection in C6/36 cells. Sequential coinfection of DENV and ZIKV in C6/36 cells was visualized using double immunofluorescence, which confirmed their presence in cells (A). The analysis of growth kinetics revealed a progressive and significant inhibition of ZIKV replication in DENV-infected cells (B). However, subsequent ZIKV infection did not have an impact on DENV replication (C). *n* = 4 samples for each group. Bars represent mean ± SEM, * *P* < 0.05 by two-tailed Mann Whitney U test.

In order to gain initial insights into the interactions between DENV and ZIKV upon sequential co-infection in mosquitoes, the mosquito C6/36 cell line was utilized for sequential co-infection of the two viruses. Results showed that C6/36 cells could be sequentially coinfected by DENV and ZIKV (Fig 1A). The growth kinetics of DENV and ZIKV was evaluated by RT-qPCR. The data revealed that at 12-, 24- and 36-hours post sequential coinfection, prior DENV infection did not significantly impact ZIKV replication (Fig 1B, *P* > 0.05). However, at later time points (48-, 60- and 72-hours post-infection), ZIKV copies were significantly lower compared to DENV-free C6/36 cells (*P* < 0.05, Fig 1B). Furthermore, subsequent ZIKV infection had no effect on DENV replication at any of the monitored time points (*P* > 0.05, Fig 1C).

### DENV infected *A*. *aegypti* has significantly decreased ZIKV vector competence

**Fig 2:**
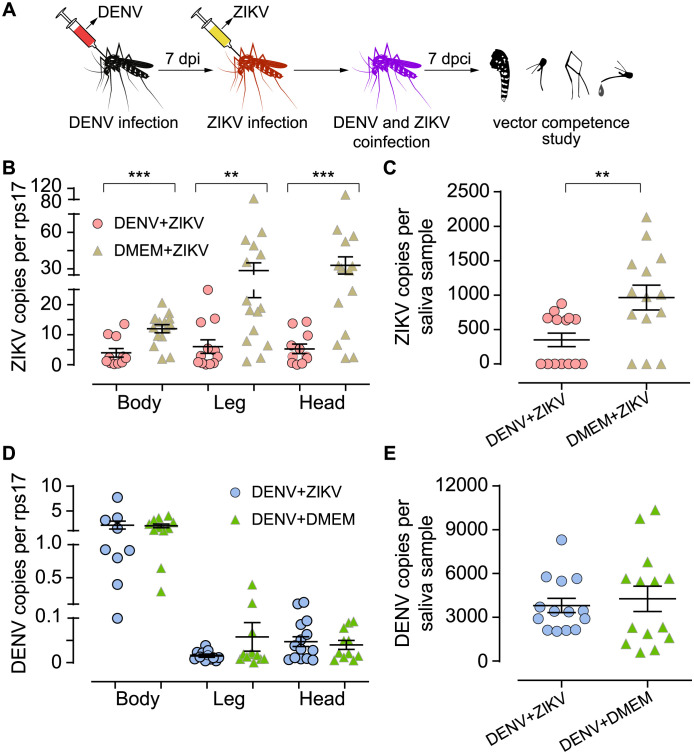
Previously DENV infected *A*. *aegypti* have significantly reduced susceptibility to ZIKV infection. *A*. *aegypti* mosquitoes were initially infected with DENV through intrathoracic inoculation and then injected with ZIKV after 7 days. The vector competence of ZIKV in *A*. *aegypti* was analyzed 7 days post coinfection (dpci). Two control groups were set as follows: (1) mosquitoes were initially injected with DMEM and later, after a 7-day interval, with ZIKV (group DMEM+ZIKV), (2) mosquitoes were initially injected with DENV and later, after a 7-day interval, with DMEM (group DENV+DMEM) (A). The results indicate that ZIKV replication, dissemination (B), and transmission (C) in DENV-infected *A*. *aegypti* are all significantly reduced. However, subsequent ZIKV infection did not influence DENV replication, dissemination (D), and transmission potential (E). *n* = 9–15 for each group. Bars represent mean ± SEM, ** *P* < 0.01, *** *P* < 0.001, and **** *P* < 0.0001 by two-tailed Mann Whitney U test. The mosquito clipart in the figure (as well as in the figures below) was obtained from OpenClipart.

To investigate if mosquitoes previously infected with DENV could have any effect on subsequent ZIKV infection, female *A*. *aegypti* were sequentially coinfected with DENV and ZIKV by intrathoracic inoculation (Fig 2A). Compared to single ZIKV infection (control group: DMEM+ZIKV), DENV-infected mosquitoes had significantly limited the ZIKV replication in the bodies (Fig 2B, group: DENV+ZIKV, *P* = 0.0008) and the ZIKV dissemination in both the legs and heads (Fig 2B, legs: *P* = 0.0014; head: *P* = 0.0009). Moreover, ZIKV copies in the saliva samples were significantly reduced (Fig 2C, *P* = 0.006). We studied if subsequent ZIKV infection had any influence on DENV development and the results showed that DENV replication, dissemination, and transmission in mosquitoes were not affected by the subsequent ZIKV infection (all *P* > 0.05, Fig 2D and 2E).

### ZIKV infected *A*. *aegypti* has significantly reduced DENV susceptibility

**Fig 3:**
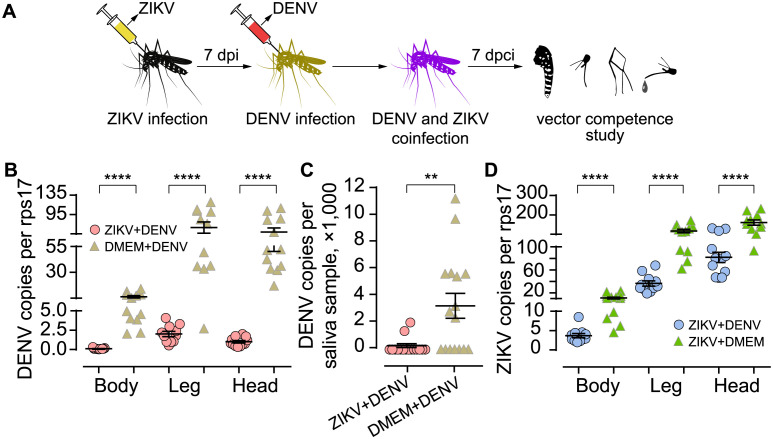
Previously ZIKV infected *A*. *aegypti* have significantly reduced DENV susceptibility. Mosquitoes were infected with ZIKV by intrathoracic inoculation, and 7 days later, DENV was injected. Then seven days post coinfection (dpci), DENV genome copies in bodies, legs, heads and saliva samples were quantified. Two control groups were set: (1) mosquitoes were initially injected with DMEM and then, after a 7-day interval, with DENV (group DMEM+DENV); (2) mosquitoes were initially injected with ZIKV and then, after a 7-day interval, with DMEM (group ZIKV+DMEM) (A). DENV genome copies in the bodies, legs, and heads, as compared to the control group (group DMEM+DENV) were all significantly lower in previously ZIKV-infected *A*. *aegypti* (group ZIKV+DENV), *n* = 10–12 for each group, **** *P* < 0.0001 by two-tailed Mann Whitney U test (B). DENV genome positive rate in saliva samples from previously ZIKV-infected mosquitoes were significantly lower, *P* = 0.0189 by Fisher’s exact test; DENV copy numbers were significantly lower as compared to the control, ** *P* < 0.01 by two-tailed Mann Whitney U test. *n* = 20 for each group (C). Succeeding DENV infection significantly affected ZIKV replication and dissemination, **** *P* < 0.0001 by two-tailed Mann Whitney U test (D). Bars represent mean ± SEM.

To investigated whether ZIKV-infected *A*. *aegypti* could also impede the development of the subsequently infected DENV, we sequentially infected *A*. *aegypti* with ZIKV and DENV via thoracic injection (Fig 3A). Results demonstrated that previous ZIKV infection in *A*. *aegypti* significantly limited DENV replication in bodies and dissemination to legs and heads (Fig 3B, all *P* < 0.0001). In addition, we observed that, in 16 tested saliva samples from prior ZIKV infected mosquitoes, only 2 sample were DENV genome positive, while in the control group (DMEM+DENV) 56.25% (9/16) saliva samples were DENV positive (Fig 3C, *P* = 0.0189), and the significant difference also presented in DENV genome copies (Fig 3C, *P* = 0.0024). We also investigated that if subsequent DENV infection could have any effect on previous ZIKV development, and the data showed that ZIKV replication and dissemination were both significantly decreased following DENV infection (Fig 3D, all *P* < 0.0001).

### DENV infected *Ae*. *aegypti* cannot transmit ZIKV under a natural virus infection approach

**Fig 4:**
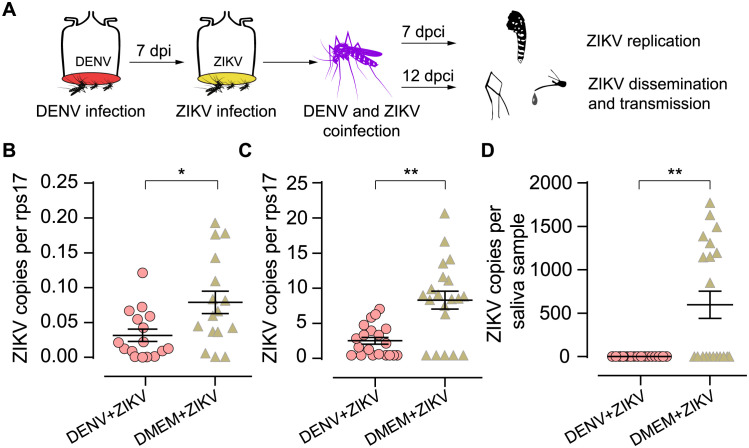
Experimental evidence of aborted Zika virus transmission in previously DENV-infected *A*. *aegypti*. Firstly, mosquitoes were infected with DENV by oral-feeding, and 7 days later, ZIKV was orally delivered to those DENV-infected mosquitoes. Seven days post-coinfection (dpci), ZIKV copies in the mosquito body, and 12 dpci, ZIKV load in saliva and legs were measured. One control group was set: mosquitoes were initially fed with DMEM-mixed blood and then, after a 7-day interval, with ZIKV (group DMEM+ZIKV) (A). ZIKV copies in the body (B) and legs (C) were significantly lower in previously DENV-infected mosquitoes. Also, ZIKV genome copies in saliva samples was significantly lower (D). *n* = 16–20 for each group. Bars represent mean ± SEM, * *P* < 0.05, ** *P* < 0.01 by two-tailed Mann Whitney U test.

To better simulate the situation of virus infection in mosquitoes, and given the higher prevalence of DENV compared to ZIKV, leading to a high likelihood of sequential coinfection with DENV preceding ZIKV in the real world, we sequentially infected *A*. *aegypti* with DENV followed by ZIKV using oral-feeding and subsequently examined the replication, dissemination, and transmission potential of ZIKV (Fig 4A). Firstly, in the DENV+ZIKV group, after 7 days of coinfection, all 16 tested mosquito bodies exhibited positive for both DENV and ZIKV infection, validating the appropriateness for examining the impact of the DENV-infected mosquitoes on ZIKV development (S1 Table). Consistent with above observations, the quantities of ZIKV copies in the bodies (Fig 4B) and legs (Fig 4C) of mosquitoes that had previously been infected with DENV were significantly reduced (bodies: *P* = 0.032; legs: *P* = 0.0014), indicating ZIKV replication and dissemination were significantly constrained in these mosquitoes. In addition, ZIKV transmission potential was significantly reduced in prior DENV-infected mosquitoes, no ZIKV genome-positive saliva samples were detected in DENV-infected *Ae*. *aegypti* (0/20), compared to 45% (9/20) of positive saliva samples in the control group (Fig 4D, DMEM+ZIKV, Fisher’s exact test, *P* = 0.001), and the ZIKV genomic copies from DENV-infected mosquitoes was also significantly lower (Fig 4D, *P* = 0.001).

### Virus infection activates mosquito immune system, perturbs lipid synthesis and enhances siRNA pathway

**Fig 5:**
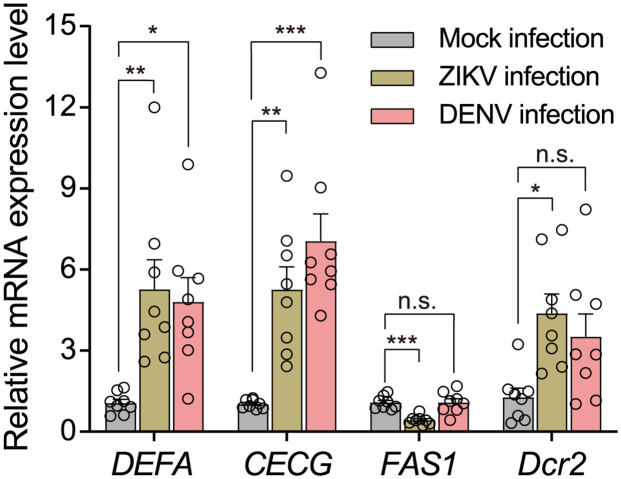
The relative mRNA expression levels of *DEFA*, *CECG*, *FAS1* and *Dcr2* in *A*. *aegypti* post DENV or ZIKV infection. Twenty-four hours post virus infection, the mRNA expression levels of related genes were quantified by RT-qPCR. The control group was mock-infected by DMEM injection. n = 8 for each group, Bars represent mean ± SEM. n.s. no significance, * *P* < 0.05, ** *P* < 0.01, *** *P* < 0.001 by one-way ANOVA with Bonferroni correction.

To gain a better understanding of the aforementioned phenomenon, we conducted preliminary investigations on the expression levels of mosquito’s antiviral-related genes (associated with innate immunity, lipid metabolism and RNAi) following DENV or ZIKV infection. Data indicated that, as compared to mock-infected mosquitoes, DENV and ZIKV infection both induced significantly higher expression levels of *DEFA* and *CECG* (Fig 5, *DEFA*: ZIKV, *P* = 0.005; DENV, *P* = 0.013. *CECG*: ZIKV, *P* = 0.002; DENV, *P* = 0), indicating mosquito innate immunity was activated by virus infection. But the expression levels of these two genes induced by DENV or ZIKV showed no significant difference (Fig 5, *DEFA*: *P* = 1; CECG: *P* = 0.327). Interestingly, we observed that ZIKV infection significantly down-regulated *FAS1* expression level (Fig 5, *P* = 0), while DENV infection did not affect it (Fig 5, *P* = 1). In addition, we observed that both DENV and ZIKV infection enhanced RNAi pathway, as suggested by the substantial increase in expression levels of *Dcr2* following virus exposure. However, it is notable that only ZIKV infection exhibited statistically significant difference (Fig 5, ZIKV: *P* = 0.011; DENV: *P* = 0.084).

## Discussion

Numerous studies have provided evidence indicating that simultaneous arbovirus coinfection in mosquitoes has the potential to promote the co-circulation of these viruses and contribute to disease epidemics [27]. However, the current understanding regarding whether sequential coinfections of arboviruses in mosquitoes similarly facilitate viral epidemics remains insufficient. Indeed, arbovirus sequential coinfection in mosquitoes can readily occur in areas where these viruses co-circulate, particularly when mosquitoes feed on individuals infected with different arboviruses during one or multiple gonotrophic cycles [46]. To address this gap in knowledge, we conducted experiments aimed at examining the phenomenon of arbovirus sequential coinfection in *A*. *aegypti*, specifically focusing on dengue virus (DENV, serotype 2) and Zika virus (ZIKV).

To gain initial insights into the interactions between DENV and ZIKV during sequential co-infection in mosquitoes, we firstly coinfected C6/36 cells with DENV and ZIKV sequentially. We observed that, C6/36 cells can support the sequential coinfection, and the prior DENV infection significantly reduced the ZIKV development, which is in accordance with previous studies performed on various cell lines with multiple viruses [47,49,66,67].

However, it should be noted that C6/36 cell lines lack a fully functional immune system, which may yield different results when studying sequential virus co-infection in mosquitoes. Considering this, we proceeded to investigate sequential co-infection of DENV and ZIKV in *A*. *aegypti* mosquitoes. Firstly, *A*. *aegypti* was sequentially infected with DENV-2 and ZIKV by intrathoracic injection. We found that *A*. *aegypti* were readily coinfected, and prior DENV infection significantly impeded the subsequent development of ZIKV, resulting in reduced dissemination of ZIKV within the mosquito and a noteworthy decrease in its transmission potential. Similarly, when mosquitoes were first infected with ZIKV by intrathoracic inoculation, subsequent DENV infection would be inhibited as well. Thoracic inoculation enables the virus to bypass the midgut barrier, facilitating efficient infection and rapid dissemination within the mosquito [68]. However, in natural conditions, mosquitoes are infected by feeding on viremic patients, therefore the virus need to overcome the midgut barrier. Considering that the antiviral system of mosquitoes and the microbiome in the midgut lumen could both strongly suppress or even abort virus infection [69–71], sequential coinfection of DENV and ZIKV in mosquitoes via oral feeding may provide a more representative depiction of real-world circumstances, as compared to the experimental scenario discussed above. Therefore, we conducted sequential coinfection of viruses through oral-feeding. Given the higher prevalence of DENV compared to ZIKV, leading to a higher likelihood of sequential coinfection with DENV preceding ZIKV in the real world, we therefore only sequentially infected *A*. *aegypti* with DENV followed by ZIKV by oral-feeding. In comparison to thoracic inoculation, we identified a similar pattern of virus interactions, but observed a stronger inhibition of ZIKV development in mosquitoes previously infected with DENV through oral feeding, where ZIKV transmission was almost aborted, as indicated by the absence of ZIKV genome in all examined saliva samples. This phenomenon could potentially be related to midgut immunity, which was stimulated by the prior virus oral infection [72]. Although we did not specifically investigate the sequential oral coinfection with ZIKV followed by DENV in this study, we can reasonably predict a higher level of DENV suppression based on these findings.

Mosquito immunity will response to virus infection [73–75]. Studies indicated that innate anti-viral pathways (associated with TOLL, IMD, and JAK/STAT pathways) may be virus-specific, but the activation of these signaling pathways may simultaneously affect multiple viruses [76–78]. In this investigation, we conducted quantitative analysis of mRNA expression levels of *DEFA* and *CECG*, two typical effector genes involved in combating viral infections [71,79,80], twenty-four hours after mosquitoes injected with DENV or ZIKV. As anticipated, we observed that both virus infection increased the expression levels of *DEFA* and *CECG*. As virus infection could modify mosquitoes’ lipid metabolism to facilitate their development [81–84], we then quantified expression level of *Fatty Acid Synthase 1* (*FAS1*), a key gene in regulating lipid metabolism, after virus infection. Interestingly we found ZIKV infection in *A*. *aegypti* could significantly down-regulate *FAS1* expression while DENV did not influence it. Based on the data from mosquito sequential coinfection by thoracic inoculation, we noticed that when mosquitoes were first infected with ZIKV, the subsequently infected DENV had significantly inhibited development: DENV copies in legs and heads have an average of 33.8-fold and 58.5-fold decrease, respectively. While in previously DENV-infected *A*. *aegypti*, ZIKV copies in legs and heads have an average of 4.5-fold and 6.2-fold decrease, respectively. The stronger DENV inhibition by previous ZIKV infection in mosquitoes may relate to the significantly downregulated *FAS1* expression, as it had been well proved that fatty acid synthesis in mosquitoes is crucial for DENV replication [81,84]. The exogenous small interfering RNA (siRNA) pathway is widely acknowledged as a pivotal component of the arthropod antiviral immune response [65,85,86], we therefore quantified the mRNA expression level of the *Dicer2* (*Dcr2*) gene, which encodes one of the key enzymes involved in RNAi pathway. We observed that both ZIKV and DENV infection induced high level expression of *Dcr2*, indicating that there was a high level of RNA-induced silencing complex (RISC) that can target and degrade the viral RNA. Taken together, the observed phenomenon that a previously flavivirus infected mosquitoes had reduced or even aborted vectorial capacity for transmitting another flavivirus may be attributed to elevated mosquito immunity and perturbed metabolism induced by the prior virus infection, thereby an antagonistic environment for arboviruses was created. However, further research is needed to clarify the underlying mechanisms.

In this study, we observed that when mosquitoes were initially infected with DENV via thoracic inoculation, subsequent infection with ZIKV did not exert any discernible influence on DENV development. However, when mosquitoes were first infected with ZIKV, subsequent DENV infection led to a substantial reduction in ZIKV replication and dissemination. The underlying mechanism responsible for this phenomenon remains elusive and may be linked to the asymmetric competition between DENV and ZIKV for host resources, a phenomenon previously documented among DENV serotypes in cell cultures [87]. However, it is worth noting that observed phenotypic virus interactions may stem from the absence of consistent validation experiments, particularly in the context of sequential oral coinfection in mosquitoes. Further investigations are warranted to elucidate the intricate interplay between these two viruses. A recent study also conducted DENV (serotype 2) and ZIKV sequential coinfection in *A*. *aegypti* [88]. One of their findings is consistent with our own observations as ZIKV-infected *A*. *aegypti* strongly suppressed the replication of subsequently infected DENV (group ZIKV → DENV compared to BM → DENV). However, when *A*. *aegypti*, previously infected ZIKV, was subsequently infected with DENV (group ZIKV → DENV), the replication of the prior ZIKV infection was significantly increased compared to the ZIKV infection alone in mosquitoes (group ZIKV → ZIKV). In contrast, our research demonstrated that the subsequent DENV infection resulted in a significant reduction in the replication of previously infected ZIKV. At present, we are unable to offer a conclusive explanation for this disparity, which could potentially stem from variations in virus genotype, virus infection routes (oral infection versus thoracic inoculation), and the method of measuring virus quantities (including virus genome copies versus the number of infectious particles). These factors should be taken into consideration to ensure precision and scientific rigor, and further emphasizes the intricate nature of virus interactions within mosquitoes following sequential coinfection.

It is noticeable that in arbovirus sequential coinfection, prior virus titer might play a critical role in interfering with later virus development [88]. In this study, the initial viruses used for thoracic inoculation were at a similar dose (DENV: 10^5.5^ copies/ml and ZIKV: 10^5.4^ copies/ml), which could minimize the density-dependent competitive suppression of the second virus. In arboviruses sequential coinfection in mosquitoes, the time interval between two infections may also influence the virus interaction [89], since the previously infected virus may have developed into abundant numbers and therefore causes density-dependent suppression effect on the latter one.

In this study, we selected a 7-day interval between two infection events, as in our laboratory settings, mosquitoes were not willing to seek a secondary virus-contained blood meal until 7 days after the first blood meal. However, it is worth noting that in natural circumstances, *A*. *aegypti* mosquitoes may search for the secondary blood meal as early as 2–4 days after the first one [90,91]. Therefore, in our experiment setup, compared to the natural scenario, previously infected viruses had an extended incubation period and potentially reached higher densities within mosquitoes, potentially leading to more pronounced inhibition of subsequent virus replication. Nevertheless, it is important to acknowledge that the dynamics of arbovirus growth in mosquitoes are intricate and influenced by various factors, including mosquito lines, virus species, temperature variations, composition of mosquito microbiota, and the initial viral infection doses. Thus, our study offers one representation of a scenario that can occur naturally, and further experimental investigations are imperative to refine and broaden upon our conclusions.

## Supporting information

S1 Data Excel spreadsheet containing separate sheets with the underlying numerical data and statistical analysis for Figures in panels 1B, 1C, 2B, 2C, 2D, 2E, 3B, 3C, 3D, 4B, 4C, 4D, and 5 of the study. (XLSX)

S1 Table The number of mosquito tissues (body and legs) or saliva samples examined to determine the infection frequency of DENV or ZIKV in DENV+ZIKV sequential oral coinfection. (DOCX)
