# Supplementary material for: Biased virus transmission following sequential coinfection of Aedes aegypti with dengue and Zika viruses
Source: PLoS Negl Trop Dis. 2024 Apr 1;18(4):e0012053. doi: 10.1371/journal.pntd.0012053 (PMC10984552; doi:10.1371/journal.pntd.0012053)
Supplement: S1 Table — (DOCX) [file pntd.0012053.s002.docx]

## Supporting information

**S1 Table**. **The number of mosquito tissues (body and legs) or saliva samples examined to determine the infection frequency of DENV or ZIKV in DENV+ZIKV sequential oral coinfection.**

|  | **DENV+ZIKV** | | | **DMEM+ZIKV** | | |
| --- | --- | --- | --- | --- | --- | --- |
|  | **Body** | **Legs** | **Saliva** | **Body** | **Legs** | **Saliva** |
| NO. of samples checked | 16 | 20 | 20 | 16 | 20 | 20 |
| NO. of ZIKV positive samples | 16 | 14 | 0 | 16 | 15 | 9 |
| ZIKV positive rate | 100% | 70% | 0% | 100% | 75% | 45% |
| NO. of DENV positive samples | 16 | n.d. | n.d. | -- | -- | -- |
| DENV positive rate | 100% | n.d. | n.d. | -- | -- | -- |

n.d. stands for not done.
